# Role of Microglial Cells in Alzheimer’s Disease Tau Propagation

**DOI:** 10.3389/fnagi.2019.00271

**Published:** 2019-10-04

**Authors:** Ena Španić, Lea Langer Horvat, Patrick R. Hof, Goran Šimić

**Affiliations:** ^1^Laboratory for Developmental Neuropathology, Department of Neuroscience, Croatian Institute for Brain Research, University of Zagreb School of Medicine, Zagreb, Croatia; ^2^Nash Family Department of Neuroscience, Ronald M. Loeb Center for Alzheimer’s Disease, Friedman Brain Institute, Icahn School of Medicine at Mount Sinai, New York, NY, United States

**Keywords:** Alzheimer’s disease, blood-brain barrier, inflammation, microglia, neurodegeneration, tau protein propagation

## Abstract

Uncontrolled immune response in the brain contributes to the progression of all neurodegenerative disease, including Alzheimer’s disease (AD). Recent investigations have documented the prion-like features of tau protein and the involvement of microglial changes with tau pathology. While it is still unclear what sequence of events is causal, it is likely that tau seeding potential and microglial contribution to tau propagation act together, and are essential for the development and progression of degenerative changes. Based on available evidence, targeting tau seeds and controlling some signaling pathways in a complex inflammation process could represent a possible new therapeutic approach for treating neurodegenerative diseases. Recent findings propose novel diagnostic assays and markers that may be used together with standard methods to complete and improve the diagnosis and classification of these diseases. In conclusion, a novel perspective on microglia-tau relations reveals new issues to investigate and imposes different approaches for developing therapeutic strategies for AD.

## Introduction

**Figure 1:**
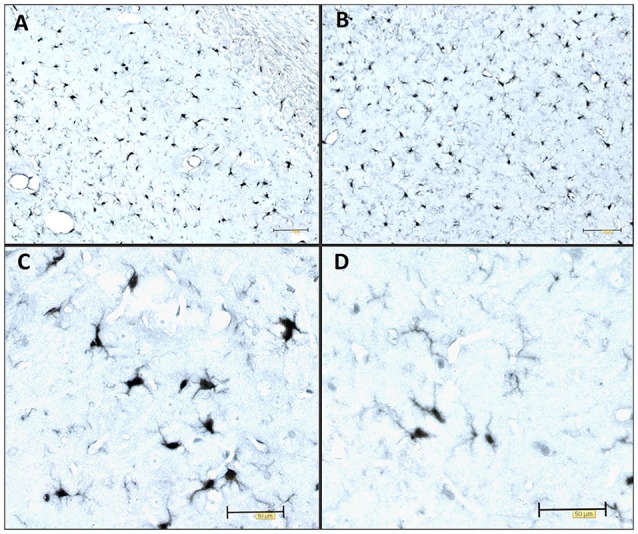
Immunocytochemical expression of the Iba1 marker visualizes the morphology of microglia. Illustrative examples of Iba1-expressing microglia in various stages of activation in the adult Wistar rat brain 3 days after inoculation of 4 μg of preformed synthetic tau fibrils (gift of Dr. Rakez Kayed, Galveston, TX, USA) into the entorhinal cortex. **(A)** Hippocampus (CA1), **(B)** entorhinal cortex, **(C)** activated microglia, **(D)** resting microglia. Scale bars **(A,B)** 100 μm, **(C,D)** 50 μm.

The most common cause of dementia is Alzheimer’s disease (AD), a progressive neurodegenerative disease that affects nearly 50 million people in the world. Histopathological features of AD are extracellular amyloid-β (Aβ) plaques and intracellular aggregation of hyperphosphorylated tau protein in a form of neurofibrillary tangles (NFTs; Braak and Braak, 1991a). The disease is also characterized by loss of neurons and synapses and elevated levels of inflammatory factors (Kinney et al., 2018). Many studies have shown the harmful effect of tau protein oligomers and its potential to propagate through synaptically connected regions (Kfoury et al., 2012; Liu et al., 2012; Plouffe et al., 2012; Smolek et al., 2019). Tau protein aggregation and neurofibrillary lesions also show the highest correlation with the clinical symptoms of the disease (Arriagada et al., 1992; Bierer et al., 1995). As such, tau protein aggregation, neurofibrillary lesions, and cytoskeletal abnormalities are considered to be a central pathogenetic mechanism of AD (Šimić et al., 1998) important for its progressive nature (Clavaguera et al., 2009; Šimić et al., 2017). Inflammation is another crucial factor that can contribute to disease progression. Neuroinflammation occurs in many neurodegenerative diseases, including AD. Several studies confirmed elevated levels of pro-inflammatory cytokines and stronger microglial activation during disease progression (Griffin et al., 1989; Lanzrein et al., 1998; Minghetti, 2005). For example, we observed Iba1-expressing microglia to be present in various stages of activation in the rat brain after inoculation of synthetic tau fibrils (Figure 1). Activated microglia have been found near NFT-bearing neurons (Sheffield et al., 2000). There is also a better correlation between numbers of activated microglia and NFT than between microglial cells’ activation and amyloid plaques distribution (Serrano-Pozo et al., 2011). Several lines of evidence suggest that inflammation may even precede the development of tau pathology (Yoshiyama et al., 2007; Denver and McClean, 2018). Therefore, controlling the components in a complex inflammatory process represents a new possible therapeutic approach for neurodegenerative diseases (for review see Šimić et al., 2019).

## Microglial Cells

Microglial cells are a population of brain myeloid cells that represent a part of the innate immunity system. Myeloid cells arise from the yolk sac and colonize the central nervous system (CNS) during early embryonic development. Their appropriate response is crucial for maintaining homeostasis and to carry out the immune response in the CNS (Ginhoux et al., 2013). Innate and adaptive immunity are the two main components of the immune response in the human body. Innate immunity is a general response, recognizing pathogen-associated molecular patterns (PAMPs) and damage-associated molecular patterns (DAMPs) through pattern recognition receptors (PRRs). In contrast, the adaptive immune response is based on the recognition of specific antigen components and acts against a specific pathogen. Adaptive immunity includes the formation of specific antibodies and involves memory cells to eliminate pathogens more efficiently upon recurrence after first exposure (Medzhitov, 2007). Based on specific gene expression microglial cells can be divided into three groups, early, pre-microglia, and adult microglia (Matcovitch-Natan et al., 2016). Microglial cells have different functions depending on their micro- and macro-environment or their developmental stage (Lavin et al., 2014; Matcovitch-Natan et al., 2016). Although, their role as immune agents during pathological conditions is still a matter of debate, microglial cells also play a key role during brain development. Through their phagocytic activity, they control the formation and depletion of the synapses, which is crucial for normal brain development. It has been suggested that synaptic pruning can be regulated by the chemokine receptor fractalkine (CX3CR1) expressed on microglia. In mouse model lacking CX3CR1, synaptic pruning was reduced, which led to the formation of immature brain and synapses (Paolicelli et al., 2011; Hoshiko et al., 2012). Furthermore, it appears that the CX3CR1/CX3CL1 complex has also a neuroprotective effect while lacking either fractalkine (CX3CL1) or its receptor leads to neurodegeneration. Elevated microglia cell activation and neuronal loss have been also noticed in SOD1G93A/CX3CR1^−/−^ mice (Liu et al., 2019). One study showed that microglia can regulate the number of neural precursor cells in the developing brain. These authors suggested that the number or activation state of microglia affect the precursor cells number and concluded that any factor that can change the number or activation state of microglia can affect neural development as well as behavioral outcomes (Cunningham et al., 2013). Depletion of the synapses is complement-dependent and it is mediated by complement receptor 3 (CR3) together with neuronal activity (Schafer et al., 2012). By releasing specific mediators that promote cell genesis, health, and dendritic growth, microglia has additional beneficial roles too (Lenz and Nelson, 2018).

In the mature brain, microglia play an important role as the main immune agents. Under physiological conditions, microglial cells are in the resting state, but they actively survey the brain parenchyma with motile processes (Nimmerjahn et al., 2005). In this state, microglia are sensitive to a wide range of stimuli like injury, toxins, pathogens, misfolded proteins, and damaged neurons (von Bernhardi et al., 2015). Similar to the polarization of macrophages in peripheral organs, it was proposed that microglia differentiate into two different phenotypes depending on their extracellular environment (Michelucci et al., 2009). The M1 or pro-inflammatory phenotype releases inflammatory mediators such as interleukin 1-β (IL-1β), tumor necrosis factor-α (TNF-α), nitric oxide (NO), reactive oxygen species (ROS), proteases, and many others in order to eliminate the potential pathogens. In contrast, the M2 phenotype is considered neuroprotective due to its phagocytic activity and anti-inflammatory effect. M2 microglia support tissue repair, reconstruction of extracellular matrix, and releases anti-inflammatory cytokines, which together promote a homeostatic environment (von Bernhardi et al., 2015; Tang and Le, 2016). Although the M1/M2 model is widely adopted, a recent study proposed a different perspective relating to the microglia disease phenotype (Keren-Shaul et al., 2017). Single-cell RNA sequencing of microglia cells from human AD brain and 5xFAD mice identified a unique subtype of microglia, called disease-associated microglia (DAM) that is characterized by a specific gene expression profile. These authors found elevated expression of some AD-related genes but decreased expression of some genes normally expressed in homeostatic microglia. Two stages in DAM activation were suggested, a triggering receptor expressed on myeloid cells 2 (TREM2) independent stage followed by TREM2-dependent activation (Keren-Shaul et al., 2017). Similar to the M1/M2, another study proposed the existence of pro-inflammatory and anti-inflammatory DAM phenotypes and the potential benefits of suppressing pro-inflammatory DAM (Rangaraju et al., 2018).

## Inflammation in AD

Neuroinflammation occurs in most neurodegenerative diseases (for review see Stephenson et al., 2018). Even though activation of microglial cells should be protective in the short-term, under altered conditions microglia sometimes promote continuous inflammation and oxidative stress which defeats homeostasis (Minghetti, 2005; Sardi et al., 2011; von Bernhardi et al., 2015; Jiang et al., 2019). In AD, microglia are supposed to be in an continuously activated state due to Aβ or some other extra- or intracellular product(s) of APP metabolism as well as due to neurofibrillary changes (Bellucci et al., 2004; Sasaki et al., 2008; Serrano-Pozo et al., 2011; Appel et al., 2014; Kametani and Hasegawa, 2018). A consequence of severe and constant microglial activation is the excessive production of pro-inflammatory mediators that creates a cytotoxic environment. Overactive microglia are associated with neuronal loss and decline of cognitive functions (Cagnin et al., 2001; Qin et al., 2002). It seems that the overproduction of ROS contributes the most to the deleterious effects of inflammation. Oxidative stress further induces production of pro-inflammatory cytokines consequently creating a vicious cycle that results in neurodegeneration (Wu et al., 2016). One study showed that microglia activation by Aβ could suppress cell proliferation and promote apoptosis of neural stem cells due to oxidative stress (Jiang et al., 2016a). In another study the same group demonstrated that activated microglia could release NO and pro-inflammatory cytokines that increased ROS levels and induced oxidative stress injury in dopaminergic neurons (Jiang et al., 2019), suggesting that harmful effects of inflammation cannot be ignored when considering potential therapeutic approaches for neurodegenerative diseases. Peripheral immune cells infiltrate the brain during neurodegeneration (Zilka et al., 2009; Sardi et al., 2011). The detrimental effect of inflammation contributes to the disruption of the blood-brain barrier in AD (Sardi et al., 2011). Therefore, a greater influx of the peripheral immune cells can occur. Although what the effects of these peripheral immune cells might be is not completely clear, it has been proposed that these cells further exacerbate inflammation (Sardi et al., 2011), while others have argued that there may be beneficial effects of the peripheral cells influx (Dionisio-Santos et al., 2019). In this context, it is interesting to note that the beneficial effect of non-steroidal anti-inflammatory drugs (NSAID) on AD incidence has been noted early in in t’ Veld et al. (2001) and Zandi et al. (2002). Apart from human studies in animal models, it has been documented that neurotrophin treatment caused a decrease in pro-inflammatory cytokines IL-1β IL-6 (TNF-α), elevation of brain-derived neurotrophic factor (BDNF), and less cognitive impairment, Aβ deposition, and microglial activation (Fang et al., 2019). Interferon-β1a (IFNβ1a) is largely used to attenuate the progression of multiple sclerosis and its anti-inflammatory effect could be beneficial also for AD patients. In an AD rat model, treatment with IFNβ1a improved memory impairment and decreased inflammation and ROS production (Mudò et al., 2019). Such possible interventions have not yet been tested in AD patients.

## Propagation of Tau Protein

**Figure 2:**
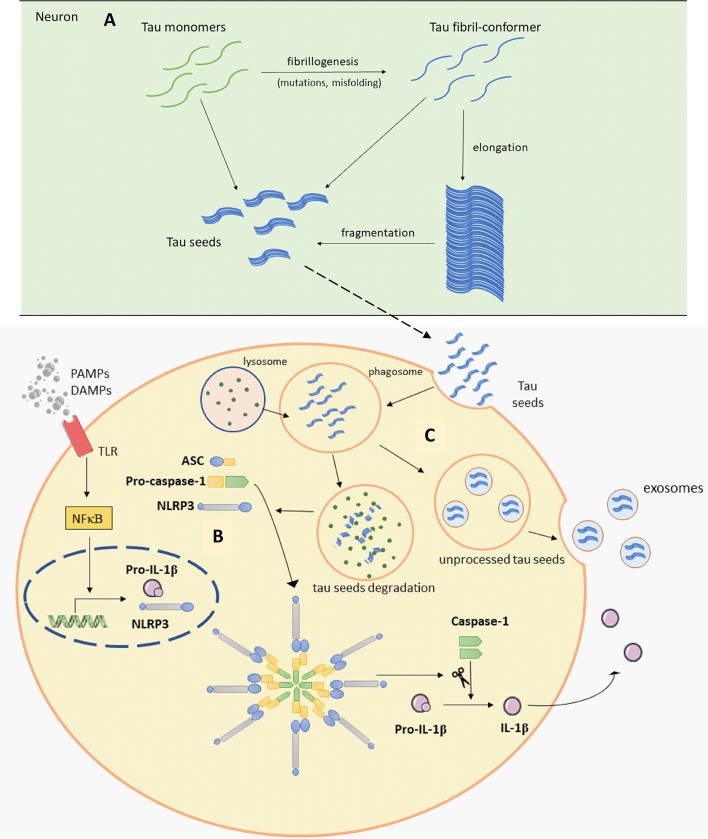
Schematic representation of Panel **(A)**. Tau seeds formation (based on Nizynski et al., 2017). **(B)** NLRP3 inflammasome activation (based on data by Stancu et al., 2019), and (**C)** microglia releasing tau seeds in exosomes (based on Hopp et al., 2018). Aβ, amyloid β; ASC, apoptosis-associated speck-like protein containing a C-terminal caspase recruitment domain (CARD); DAMPs, damage-associated molecular patterns; mtROS, mitochondrial reactive oxygen species (ROS); NLRP3, NACHT, LRR and PYD domains-containing protein 3; PAMPs, pathogen-associated molecular patterns; TLR, toll-like receptor. See text for details.

Tau protein is mainly expressed in neurons and is essential for microtubule assembly and stabilization of the cytoskeleton (Weingarten et al., 1975). Tau is encoded by the *MAPT* gene and different RNA splicing can produce six tau isoforms in the human brain. Isoforms of tau have either three or four microtubule-binding repeats (Neve et al., 1986; Goedert et al., 1989). Phosphorylation is one of the main posttranslational changes of tau and is crucial for the regulation of tau functions as it can reduce its ability to bind to microtubules (Lindwall and Cole, 1984). Therefore, only the correct pattern of tau phosphorylation can be effective. It was proposed that hyperphosphorylation can cause abnormal folding of tau. This is also supported by *in vitro* findings, where we showed that exhaustive tau phosphorylation is either not essential for the stability of the putative tau oligomer once it is formed, or is necessary for initial oligomer formation (Boban et al., 2019). Once formed, tau oligomers can be stable even upon dephosphorylation (Boban et al., 2019), but misfolded tau is not able to stabilize microtubules properly, consequently causing its degradation and intracellular self-aggregation. Thus, the accumulation of abnormally phosphorylated tau is one of the earliest changes in the process of NFT formation (Bancher et al., 1989; Braak et al., 1994; Šimić et al., 2016). Because of the positive correlation between NFT formation and loss of cognitive function (Arriagada et al., 1992; Bierer et al., 1995) and the specific patterns of disease progression, the disease is usually described in terms of the Braak and Braak stages. This topographic progression of the disease is classified into six stages. The first two stages are called transentorhinal because of the progression from the entorhinal region to the hippocampal formation. Further, in stages III and IV, the disease spreads to the temporal, frontal and parietal neocortex and finally, in late stages V and VI, sensory and motor areas of the neocortex are affected (Braak and Braak, 1991b). The pathogenic spread hypothesis can explain the characteristic spread of tau, but alternative mechanisms about selective vulnerability should also be considered (Walsh and Selkoe, 2016). Both mechanisms may be acting together and contribute to spatial distribution of protein aggregates. The concept of selective neuronal vulnerability refers to a situation where certain neurons are more vulnerable than others to pathogenic processes that cause the misfolding and aggregation of tau proteins. This is perhaps determined by biochemical, genomic, connectomic, electrophysiological, and morphological properties of vulnerable neurons (Šimić et al., 2017, 2019) and region-specific microenvironments (Jackson, 2014). There are other possible reasons for vulnerability due to differential protein expression: for example, in regions that are always affected by tau pathology, elevated expression of some proteins that co-aggregate with tau and Aβ, with lower expression of proteins that can prevent aggregation of tau and Aβ has been observed, when compared to regions less susceptible to the disease (Freer et al., 2016). Recent evidence suggests that abnormal forms of tau proteins appear in a small number of neurons and propagate to other regions inducing disease progression in a prion-like manner (Clavaguera et al., 2009; Hall and Patuto, 2012; Walker and Jucker, 2015). Prions, or proteinaceous infectious particles, represent the misfolded prion protein that causes several neurological diseases known as transmissible spongiform encephalopathies (Prusiner, 1982, 2013). Prions are considered infectious due to their potential to induce misfolding of normally folded PrP molecules in neighboring cells. It is likely that prions are transmitted between cells through synaptic or vesicular transport (Aguzzi and Rajendran, 2009). Until recently, it was believed that only cell-autonomous mechanisms are responsible for sporadic neurodegenerative disease development. Cell-autonomous mechanisms imply that protein aggregation emerges independently in several cells. It has however been shown that first local inclusion propagates towards healthy cells *via* non-cell-autonomous mechanisms, which leads to degeneration (Goedert et al., 2017). For pathogenic spread of tau proteins there are few initial steps that have to occur. It has been proposed that initially a tau-competent monomer is formed. This means that such specific conformation of monomer is competent to assemble into nuclei, a supposedly rate-limiting stage for the rapid growth of tau fibrils. Elongation of the tau-competent conformers or fragmentation of tau aggregates/fibrils may produce short fragments (“seeds”), which are able to act as templates and thus recruit native tau monomers to rapid growth of tau aggregates (Figure 2A), thus skipping a long early, rate-limiting, stage (Nizynski et al., 2017). There are many experimental animal and *in vitro* studies that describe the trans-synaptic spread of tau along anatomically connected brain regions. One study demonstrated this kind of propagation in transgenic mice that differentially express pathological human tau in the entorhinal cortex (Liu et al., 2012). Furthermore, injection of insoluble tau isolated from human AD brain induces neurofibrillary pathology in rats expressing non-mutated truncated tau (Smolek et al., 2019), as well as in several non-transgenic mouse models (Clavaguera et al., 2013; Boluda et al., 2015; Guo et al., 2016; Gibbons et al., 2017; Narasimhan et al., 2017). When protein aggregates were injected into specific mouse brain region pathology progressed only into brain regions synaptically connected to the injected site, leaving the closer but unconnected neurons unaffected (Clavaguera et al., 2013). *In vitro* studies also support the prion-like feature of tau (Kfoury et al., 2012; Plouffe et al., 2012). Using an ultrasensitive FRET-based flow cytometry biosensor assay, tau seeding was detected 4 weeks earlier than other pathological tau species, marked with common histological markers in P301S mouse (Holmes et al., 2014). The same technology detected tau seeding activity in some regions that usually lack pathological tau detected with histological markers in human postmortem AD brains. The majority of Braak stage 1 samples had seeding activity in the hippocampus and some of them in some neocortical regions although histological markers of tau pathology are limited to the entorhinal cortex in stage 1. Tau seeding was found frequently in frontal and parietal lobes of stage 2 cases, where tau pathology is generally restricted to the limbic system. Some stage 3 samples exhibited tau seeding even in the cerebellum which is not typically affected by tau pathology. This suggests that seeding activity precedes other histological markers of pathological tau and could be the earliest indication of tau pathology. This assay may thus be used together with standard neuropathological methods to refine the classification of tauopathies (Furman et al., 2017). To test whether there is a direct trans-synaptic spread of protein aggregates we need to restrict aggregated protein expression to one regional population of neurons and demonstrate conversion of natively folded tau protein in synaptically connected neurons. Hyperphosphorylation and misfolding of tau can enhance neuronal tau uptake (Takeda et al., 2015; Šimić et al., 2016). Takeda et al. (2015) detected soluble phosphorylated high-molecular-weight (HMW) tau species as tau with the greatest ability to propagate in a prion-like manner. Using differential centrifugation and size-exclusion chromatography they isolated propagating tau and then assessed its uptake. Tau uptake was present only in neurons treated with extracts that contained HMW tau. Further characterization revealed small amounts of HMW tau in those extracts and only small globular tau aggregates in HMW tau fraction (Takeda et al., 2015). Tau from brain intestinal fluid (ISF) and cerebrospinal fluid (CSF) of mouse model applied on the cell culture is taken up by neurons where it can promote the formation of intracellular aggregates. As the CSF from AD patients also contains HMW tau, these results provide evidence that extracellular tau from the diseased brain can be bioactive and that HMW tau has a major seeding potential because even small amounts of bioactive HMW tau can promote its further propagation (Takeda et al., 2015, 2016). Can be taken up by neurons in culture.

## Tau Release *via* Exosomes

The prion-like hypothesis of tau transmission proposes that misfolded tau acts as a seed causing further misfolding of proteins inside healthy cells, implying that tau needs to be released outside of the cells. Tau released in the ISF can induce stronger microglial activation which in turn may promote the propagation of tau pathology. It is well known that the CSF levels of tau are elevated in AD patients (Zetterberg and Blennow, 2013; Babić et al., 2014; Blennow et al., 2015; Babić Leko et al., 2018), and this was also confirmed in tau transgenic mice model (Barten et al., 2011). One study detected tau in the ISF of wild-type mice, suggesting that tau is released into the ISF under normal conditions (Yamada et al., 2011). These investigators also tested tau levels in P301S transgenic mice. Tau levels were approximately 5-fold higher in P301S brain than in wild-type and similar differences were observed in ISF tau levels. This results proved that intracellular and extracellular tau levels are related (Yamada et al., 2011) and even though tau is predominantly a cytosolic protein, it can be actively secreted outside of the cell. Tau does not have a signal peptide for the secretory pathway, which means it cannot be released through the endoplasmic reticulum (ER)-Golgi secretory pathway. Several studies have proposed possible mechanisms of tau release and uptake. Some mentioned tau can be secreted *via* unconventional secretory pathways like other proteins lacking signal peptides (Rabouille et al., 2012; Katsinelos et al., 2018). One of the mechanisms described is tau secretion *via* exosomes. This mechanism was demonstrated in cell cultures *in vitro* such as M1C (Saman et al., 2012), N2a, and also in primary neurons (Wang et al., 2017). Exosome release is regulated by cell activity and depolarization of neurons promotes the release of tau-containing exosomes. Also, another study showed that synaptic connection is crucial for tau transmission *via* exosomes (Wang et al., 2017) which supports the possibility that synaptic connections are the main factor that influences the specific way of tau spreading and disease progression across brain regions. Exosomes isolated from the CSF of AD patients and control subjects contain tau (Saman et al., 2012; Wang et al., 2017). It is interesting that exosomes from AD and control CSF can promote aggregation of tau protein in cultured cells. Exosomes from AD patients induces slightly higher, yet statistically not significant, aggregation (Wang et al., 2017). Based on exosomes-associated tau presence in the CSF it is of interest to examine the diagnostic potential of CSF exosomes. Levels of blood exosomal tau are higher in AD patients than in control subjects (Fiandaca et al., 2015). Higher sensitivity and therefore better diagnostic potential of exosomal tau was reported compared to statistically significant tau levels evaluated only from fluid-phase. More importantly, elevated exosomal levels of tau, together with Aβ_1–42_ levels were detected up to 10 years prior to clinical diagnosis (Fiandaca et al., 2015). Exosomes and other extracellular vesicles, therefore, represent potential new biomarkers for AD and could help predict the course of the disease (Fiandaca et al., 2015). As it stands, we are still lacking standardized protocols for isolation or characterization of extracellular vesicles for clinical purpose (Gámez-Valero et al., 2019).

## Microglia-Tau Relationships

It is well known that tau can mediate an inflammatory response in neurodegenerative disease. Many studies show microglial activation upon tau pathology (Bellucci et al., 2004; Sasaki et al., 2008; Zilka et al., 2009, 2012). Activated microglia were found near NFT-bearing neurons (Sheffield et al., 2000). Also, there is a better correlation between the number of activated microglia and NFT than between glial activation and plaques distribution (Serrano-Pozo et al., 2011). In a transgenic rat model, reactive microglia were associated with neurofibrillary changes and was absent within regions with smaller neurofibrillary degeneration (Zilka et al., 2009). It was recently hypothesized that inflammation even precedes the development of tau pathology (Sheffield et al., 2000; Yoshiyama et al., 2007; Ghosh et al., 2013). In a mouse model of tauopathy, microglial activation and synaptic pathology were identified as the earliest manifestations of neurodegeneration. These authors suggested that activated microglia exacerbate tau pathology by damaging dendrites and axons. This study also provided evidence that inhibition of the inflammatory response may ameliorate consequences of tau pathology (Yoshiyama et al., 2007). Experiments in various transgenic mice models demonstrated the importance of proper microglial response in maintaining healthy brain environment. High levels of pro-inflammatory cytokine IL-1 exacerbate tau phosphorylation (Li et al., 2003; Ghosh et al., 2013). Elevated tau phosphorylation and aggregation were noticed in the absence of TREM2 or fractalkine receptor (Bhaskar et al., 2010; Maphis et al., 2015; Bemiller et al., 2017). Tau binding to CX3CR1 initiate its uptake and absence of CX3CR1 resulted in altered uptake and degradation of tau by microglia. It is proposed that tau competes with CX3CL1 to bind to this receptor (Bolós et al., 2017). Overexpression of TREM2 improves cognitive impairments, attenuates synapse loss and tau hyperphosphorylation (Jiang et al., 2016b). Another interesting study shows the different effect of partial and complete depletion of TREM2 on the progression of tau pathology, pointing out worse outcome in the case of partial TREM2 depletion (Sayed et al., 2018). These studies emphasize the importance of TREM2 and CX3CR1 signaling pathways in regulating microglial response and tau pathology. Controlling those signaling pathways outlines possible future therapeutic strategies. Furthermore, inflammatory factors from microglia cells cause upregulation of tau protein *in vitro*. Interestingly, microglia pretreated with NSAID attenuated tau overexpression (Lee et al., 2015). Microglia can actively phagocytize tau (Asai et al., 2015; Bolós et al., 2015; Luo et al., 2015; Hopp et al., 2018). Depending on tau isoform or microglial response, internalized tau can be processed in different ways. Microglia-mediated degradation of tau can be enhanced by an anti-tau monoclonal antibody (Luo et al., 2015). Moreover, if microglia fail to process tau completely into a non-toxic form, tau seeds can be released within exosomes making microglia competent to promote tau seeding in adjacent cells (Figure 2C; Hopp et al., 2018). Asai et al. (2015) tested the role of microglia in tau propagation *in vitro* and *in vivo*. AT8 and PHF1 tau colocalized within hippocampal Iba1-expressing microglia, which convincingly demonstrate microglial uptake of tau (Asai et al., 2015). *In vitro* test supported this conclusion, showing more efficient phagocytic activity by microglia when compared to neurons and astroglia (Asai et al., 2015). Activation of microglia also induced tau ubiquitination (modification important for exosomal incorporation of tau) and secretion of exosomal tau. An exosomal fraction from microglia applied on pyramidal neurons in culture or injected in the mouse brain caused neuronal uptake of exosome derived tau. Finally, it has been demonstrated that depletion of microglia and inhibition of exosome synthesis reduce tau propagation *in vitro* and *in vivo* (Asai et al., 2015).

## Tau Seeds Induce Inflammasome Activation

A recent study revealed one possible molecular mechanism that can explain noticed microglial changes in response to tau pathology and microglial contribution to tau propagation (Figure 2B; Stancu et al., 2019). Aggregated tau acting as prion-like seeds activate NLRP3-ASC inflammasome. Nod-like receptors (NLR) are one of the sensors important for activation of innate immune response. Molecular binding to NLRP3 induces the formation of ASC heteromer which further causes microglial activation and elevation of pro-inflammatory cytokine production. Stancu et al. (2019) propose that activation of NLRP3-ASC inflammasome follows microglial uptake of tau, therefore in such way inflammasome activation can promote further exogenously and non-exogenously tau seeded pathology. In ASC deficient microglia, tau-induced IL-1β secretion was blocked and in mice lacking ASC, tau pathology was significantly reduced compared with mice expressing ASC. Using NLRP3 inhibitor MCC950, they also confirmed ASC formation is dependent on NLRP3 activation (Stancu et al., 2019). Prior research by Franklin et al. (2014) revealed that ASC accumulates outside of the cells consequently inducing further IL-1β elevation. In addition, microglial uptake of ASC causes its activation, but it also can promote damage inside the cells. Based on these observations, it appears that the inflammasome can be activated in neighboring cells not just because of the tau seeds but also because of extracellular ASC heteromer uptake which creates a vicious cycle promoting constant microglial activation and severe inflammation that spreads within affected areas (Franklin et al., 2014; Stancu et al., 2019).

## Conclusions

The harmful effect of tau protein oligomers and its potential to propagate through synaptically connected regions has been well established by recent evidence, along with microglial changes in close vicinity of tau pathology. Uncontrolled microglial response in the brain contributes to the progression of many neurodegenerative diseases and several lines of evidence suggest that inflammation may even precede the development of tau pathology in AD. Exosomes could be an important link between tau propagation and microglial activation. Reduction of microglial cells number and exosome synthesis inhibition reduces tau propagation (Asai et al., 2015). Further, phagocytosed tau seeds induce inflammasome activation inside microglia causing overactive microglia state. That could be one of the mechanisms that promote the constant inflammatory response in AD. Based on the recent results presented in this mini-review, we concluded that seeding potential of tau protein and microglial activation are probably acting together and contribute to tau propagation, which could be crucial for the development and progression of degenerative changes. In conclusion, a novel perspective on microglia-tau relations reveals new issues to investigate and imposes different approaches for developing therapeutic and preventative strategies.

## Ethics Statement

All animal experiments were conducted according to the Principles of Laboratory Animal Care and ARRIVE guidelines^1^, with the approval of the Ethical Committee of the University of Zagreb Faculty of Science (EP 02/2015 from 15th August 2015) and in accordance with relevant laws (Animal Welfare Law 135/06 and 37/13) and regulations of the The Ministry of Agriculture of The Republic of Croatia (approval no. NP-999/15-01/15) from 12th October 2015).

## Author Contributions

GŠ conceived the manuscript. All authors edited the drafts of the manuscript.

## Conflict of Interest

The authors declare that the research was conducted in the absence of any commercial or financial relationships that could be construed as a potential conflict of interest.

## Footnotes

^1^http://www.nc3rs.org.uk/page.asp?id=1357
